# Transporter and Lysosomal Mediated (Multi)drug Resistance to Tyrosine Kinase Inhibitors and Potential Strategies to Overcome Resistance

**DOI:** 10.3390/cancers10120503

**Published:** 2018-12-10

**Authors:** Daniel J. de Klerk, Richard J. Honeywell, Gerrit Jansen, Godefridus J. Peters

**Affiliations:** 1 Laboratory Medical Oncology, Amsterdam UMC, VU University Medical Center, P.O. Box 7057, 1007 MB Amsterdam, The Netherlands; d.j.deklerk@amc.uva.nl (D.J.d.K.); r.honeywell@vumc.nl (R.J.H.); 2 Rheumatology and Immunology Center—Location VUmc, Amsterdam UMC, P.O. Box 7057, 1007 MB Amsterdam, The Netherlands; g.jansen@vumc.nl

**Keywords:** tyrosine kinase inhibitors, multidrug resistance, ABC transporters, lysosomes

## Abstract

Tyrosine kinase inhibitors are a class of chemotherapeutic drugs that target specific protein kinases. These tyrosine kinase inhibitors constitute a relatively new class of drugs which target for instance Bcr-Abl, Epidermal Growth Factor Receptor (EGFR) and Vascular Endothelial Growth Factor Receptor (VEGFR). Despite some initial successes, the overall therapeutic benefit of tyrosine kinase inhibitors in the clinic has been mixed. Next to mutations in the target, multidrug resistance is a major obstacle for which still no clinically effective strategies have been developed. Major mechanisms of multidrug resistance are mediated by drug efflux transporter proteins. Moreover, there is accumulating evidence that multidrug resistance can also be caused by lysosomal sequestration of drugs, effectively trapping tyrosine kinase inhibitors and preventing them from reaching their target. Lysosomal drug sequestration seems to work together with ATP-binding cassette transporters, increasing the capacity of lysosomes to mediate sequestration. Both membrane efflux transporter proteins and lysosomes present potential therapeutic targets that could reverse multidrug resistance and increase drug efficacy in combination therapy. This review describes both mechanisms and discusses a number of proposed strategies to circumvent or reverse tyrosine kinase inhibitor-related multidrug resistance.

## 1. Introduction

Tyrosine kinase inhibitors (TKI) represent a class of chemotherapeutic drugs that target protein kinases as a means of disrupting cell division and other cellular processes that contribute to tumour cell progression and proliferation. Although TKIs are a relatively new class of drugs, many are now standard therapy for the treatment of certain types of cancer. Particularly cancers that exhibit dependence upon specific oncogenic drivers, such as EGFR, ERBB2/HER2, ALK and mutant BRAF, are being treated with TKIs. Targeting these protein kinases presents a means of targeting tumour cells by blocking these processes. Protein kinases are generally classified based upon the specific amino acid residues being phosphorylated. For the treatment of cancer, TKIs are commonly used, as tyrosine kinases are involved in signaling pathways regulating proliferation [1].

Although there has been some success in the clinical use of TKIs, particularly with treatment of both chronic and acute myeloid leukemia [2,3], overall success of TKIs has been mixed with some moderate to poor survival benefits for patients [4,5,6]. In many cases, tumours acquire resistance during TKI treatment, usually between periods of eight months to a year [7,8,9,10]. In addition, intrinsic resistance can also limit the initial response or prevent drugs reaching their targets due to hurdles negatively influencing the pharmacology of the drug, such as patient specific metabolism, rapid clearance or not being able to pass through membranes of cancer cells or other barriers like the blood brain barrier [11]. Cancer cells, especially those resistant against multiple drugs, remain a major obstacle in effective cancer therapy.

With respect to TKIs, it is well recognized that (single agent) drug resistance can develop through mutations in the TKI target itself, the target pathways or alternative pathways that circumvent the mechanism of action (MoA) of a specific TKI [11]. A common strategy to this type of resistance is the development of another TKI targeting this mutations or against a different target, preferably further downstream. More difficult forms of drug resistance include multidrug resistance, often caused by transporter proteins [12], which mediate the influx and efflux of drugs. Research on these transporters has resulted in a greater understanding of the molecular mechanisms conferring drug resistance. Insight in protein structures of drug efflux transporters like P-glycoprotein (MDR1 or P-gp) has led to the development of inhibitors, which, unfortunately, were unsuccessful in a clinical setting [13]. Another, more recently discovered mechanism of multidrug resistance is based on lysosomal sequestration of drugs [14,15], which involves trapping hydrophobic drugs such as TKIs in cell organelles like lysosomes, preventing them from reaching their targets. This review provides an overview of multidrug resistance for TKIs with specific focus on the role of influx/efflux transporter proteins and lysosomal drug sequestration, including possible modalities of circumventing drug resistance.

## 2. Known Resistance Mechanism to TKI

### 2.1. Mutations and Redundant Branches in Target Signalling Pathways

**Figure 1:**
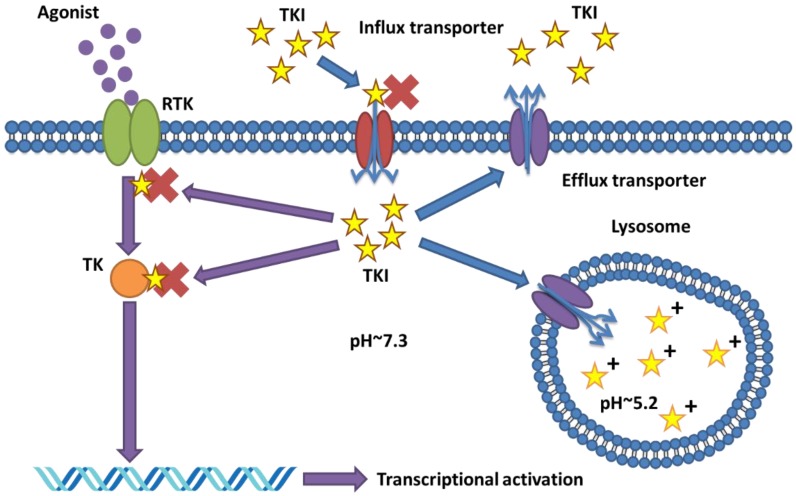
General overview of the mechanism of action and resistance for TKIs (Tyrosine kinase inhibitors). Receptor tyrosine kinases (labelled RTK) activate tyrosine kinases (labelled TK) downstream leading to transcriptional activation. Most cases of resistance involve punt mutations in the drug target (represented by the ×). Alternatively, TKI can be sequestered in the lysosome and protonated (indicated by the +) or removed from the cell through efflux transporters.

Mutations or other somatic alterations that induce resistance to TKIs are commonly located within the pathways that the TKI targets (Figure 1). For example, a documented alteration that induces resistance to EGFR inhibitors includes the T790M point mutation, which is a common mutation in the kinase domain of EGFR and linked to erlotinib, gefitinib and afatinib resistance [16,17]. Other alterations are amplifications of MET, IGF-1R, KIT, HER2 and HER3, which act as parallel pathways, activating the PI3K and RAS signaling cascades, thus circumventing EGFR inhibition [18,19,20].

These examples of acquired resistance occur after TKI treatment. However, inherent resistance to TKIs occurs via different mechanisms, for example when the TKI target is non-essential to tumour progression. Mutated Ras will lead to a constitutively activated Ras pathway, bypassing EGFR inhibition. Alternatively apoptotic resistance prevents inhibition from inducing cell death. Tumours that have the wild type EGFR are resistant to EGFR-directed TKIs, such as erlotinib and gefitinib [11]. Another example of an inherent TKI resistance specifically (but not exclusively) to EGFR is a germline mutation in BIM, resulting in reduced expression. BIM is a member of the Bcl-2 family of pro-apoptotic genes [21,22]. TKIs upregulate BIM as part of their mechanism of action through inhibition of MAPK pathway leading to apoptosis [23].

### 2.2. Role and Alterations of Transporter Proteins in TKI Resistance

In addition to somatic and germline mutations in relevant kinases, research has shown that absorption, disposition, metabolism and excretion (ADME) also plays an important role in TKI resistance [12]. Cell membrane transporter proteins mediate the influx and efflux of TKI compounds, and thus will determine the retention of TKIs and their ability to reach their target (Figure 1) [24]. In general, two families of transporters are considered as most relevant for small molecule drug-uptake, i.e., the solute carriers (SLC), which mediate drug influx and the ATP-binding cassette (ABC) family that mediates drug efflux.

For TKIs, there is no conclusive evidence to what extent the SLC family of transporters is primary responsible for uptake or in drug resistance. Reduced expression of the influx transporter OCT-1 (SLC22A) has been implicated in resistance to imatinib [12]. Another study involving CML patients resistant to imatinib also reported decreased OCT-1 expression compared to imatinib-naïve CML patients, but concomitantly pointed to a contributing role of upregulation of efflux transporters ABCB1 (P-gp) and ABCG2 (BCRP) in imatinib resistance [25]. A recent systematic review has put the relevance of OCT-1 into question [26], pointing to the lack of a specific activity assays and experimental limitations. This results in a high variation among the different studies and moderates OCT-1 as a clinically relevant predictor of innate resistance.

Most TKIs are substrates for common ABC efflux transporters, notably ABCB1 and ABCG2, while some TKIs are also substrates for some other members of the ABC family [12]. Transporters of the ABC family have been investigated extensively and are implicated in multidrug resistance. Polymorphisms within the *ABCB1* gene have presented with conflicting outcomes although there may be a role for drug response and adverse effects [27,28,29]. ABCB1 and ABCG2 are expressed in cells of relevant tissues such as intestinal lumen and blood-brain barriers, where they transport compounds back into the blood or lumen, while there have been other studies that showed an upregulated expression of these transporters during treatment [24,28,29].

### 2.3. Current Strategies to Overcome Resistance in TKI Based Therapy

To bypass drug resistance in the clinic, various approaches have been initiated. Clinical resistance to imatinib in the treatment of CML can be caused by various mutations that have been identified, such as one in the gate-keeper ABL (T3151) [29,30,31,32]. Several drugs including dasatinib and nilotinib have been developed to reverse one of 15 common imatinib resistance-related mutations in the Bcr-Abl fusion protein occurring in 85% of patients [30,32,33,34].

NSCLC resistance to EGFR inhibitors generally occurs either via the T790M de-sensitizing mutation or the so-called ‘oncogene kinase switch’, where an alternative tyrosine receptor kinase or pathway becomes the primary oncogenic driver instead of EGFR [35,36]. Resistance can also be reversed by TKIs that either target EGFR containing the T790 mutation (osimertinib) or by inhibitors for MET (crizotinib) or IGF-1R [37]. Unfortunately patients will also develop resistance to osimertinib by mutations in the EGFR active site (C797S) [36,38]. Fourth-generation EGFR inhibitors such as EA1045 are currently being developed to bypass this resistance [36,39].

Another example of resistance to TKI in NSCLC is the development of multiple inhibitors against the ALK-EML fusion protein. These patients are usually being treated with crizotinib [40]. When patients develop resistance, several alternatives are currently available such as ceritinib, alectinib, brigatinib and lorlatinib (reviewed in [40,41]). The development of these drugs is a nice example of a rationale design of an inhibitor, since they can bypass several mutations such as the steric hindrance caused by the L1196M mutation. Moreover, in contrast to crizotinib, these drugs cannot only pass the blood-brain barrier, but are not transported out of the brain by P-gp or BCRP, accumulate in the brain and are effective against brain metastases [41,42]. However, the bioavailability of lorlatinib can be affected by inhibition of P-gp [43]. Because of these properties, alectinib is now considered as a first-line therapy for adenocarcinoma NSCLC with the ALK-EML4 fusing protein [44].

A common approach to reverse drug resistance is the use of combinations. Earlier, we reported a mechanism-based approach to develop combinations for cytotoxic drugs, which led to the clinical use of combinations such as of 5-fluorouracil and leucovorin and of cisplatin with gemcitabine [45]. This involved the application of the combination index [46], which was translated to the in vivo models and the clinic [45]. A similar approach was used to design the combination of erlotinib and crizotinib, in which crizotinib mediated inhibition of the cMet pathway can bypass the resistance to erlotinib [36]. An alternative may be the so-called feedback system control, but this did not yet proceed beyond the in vitro testing phase [47].

### 2.4. Hurdles in Overcoming Resistance to TKI

Notably, other TKI-related drug resistance mechanisms pose more complicated obstacles. Transporters, especially the Multidrug resistance proteins like ABCB1 and ABCG2, confer drug efflux mediated resistance and is more challenging to circumvent [12]. Among the many compounds that have been developed to block efflux transporters [48], some TKIs themselves also exhibit the ability to reverse resistance in MDR-overexpressing cells and thus can potentially act as sensitizers in combined therapy with other TKIs [49,50,51].

## 3. Molecular Changes of Transporter Proteins in Drug Resistance

### 3.1. General Overview of the Transporters Involved in Cellular Uptake and Extrusion of TKIs

Active transporter proteins involved in cellular uptake and extrusion of TKIs include members of the SLC and ABC super families, which are ubiquitously expressed throughout human tissue [52]. Specifically, for TKI influx, the members of the SLC22 family, the organic anion and cation transporters (OAT and OCT) and the organic anion transporting polypeptides (OATP) are most relevant. TKI efflux is largely facilitated by ABCB1 (P-gp), ABCG2 (BCRP), ABCC1 (MRP1) and ABCC2 (MRP2). An overview of currently clinically approved TKIs (including FDA, EMA, and CFDA), potentially interesting TKIs in clinical development and their interactions with various influx and efflux transporters are summarized in Table 1 [50,53,54]. Although TKIs are usually considered as targeted drugs, the majority does not hit one target, but multiple targets [55]. The advantage of a multitargeted TKI is that hitting more than one target can bypass an alternative pathway, while the drug can be optimized for its ADME properties such as not being a substrate for transporters or a CYP enzyme [55]. A recent example is anlotinib, which inhibits VEGFR1, VEGFR2/KDR, VEGFR3, c-kitPDGFR1 and FGFR [53], which is an example of Chinese developed drugs, being registered for treatment of NSCLC and soft tissue sarcoma [53,54].

The OATP family consists of transporter proteins that operate in an ATP and sodium independent fashion; they rely upon the efflux of a counter ion or proton as a driver of active transport [72,73,74,75]. Most OATPs have a ubiquitous expression in epithelial and endothelial cells, but some are organ specific. OATP1A2 has a ubiquitous expression in human endothelial cells, whereas OATP1B1 and OAP1B3 are liver specific [76]. In cancer tissues, OAT1A2 is downregulated in colon cancer and gliomas [77,78], whilst upregulated in bone and prostate cancer [79,80]. Both OATPB1 and OATP1B3 are downregulated in liver cancer [81], whilst upregulated in colon and ovarian cancer [82,83]. In addition, OATP1B3 is also upregulated in breast, lung, pancreatic and prostate cancer [66].

The OAT family of transporter proteins operates like the OATP family of proteins via an efflux driven mechanism using counter ions in the form of carboxylic acids; for OAT1 and OAT3, this is α-ketoglutarate [76]. *OAT1* mRNA expression in humans has been found primarily in the kidneys and to a lesser degree in skeletal muscle, brain and placenta [84]. OAT1 protein expression has been found in the basolateral membrane of proximal tubules and plasma membrane of the skeletal muscle [85,86]. *OAT2* mRNA has been detected in both liver and kidney tissue and protein expression has been found in the basolateral membrane of proximal tubules [87,88,89]. OAT3 mRNA and protein expression has been observed in the kidney [90], *OAT3* mRNA expression in the brain and OAT3 protein expression in the adrenal tissue [88,91].

The OCT family can be divided between organic cation transporters (OCT1, OCT2 and OCT3) and organic cation and carnitine transporters (OCTN1 and OCTN2) [76]. The organic cation transporters act as passive bidirectional transporters using the exchange of cations along their electrochemical gradient. Although sodium and pH independent, the ionization-dependency implicates that the pH can affect substrate affinity [92]. The OCTNs are affected by both sodium and proton gradients, depending on the substrate [76]. mRNA of *OCT* family members of transporters can be found in most epithelial and endothelial cells [93,94]. OCT1 has been identified at the protein level in the basolateral membrane of the enterocytes and hepatocytes [76,95,96]. OCT2 is expressed at the protein level in basolateral membrane of the renal proximal tubules [97]. OCT3 protein expression has been identified in the basolateral membrane of hepatocytes [98], the apical membrane of enterocytes and the basolateral membrane of the renal proximal tubules [97,99]. OCTN1 and OCTN2 protein expression have been found at the apical membrane of enterocytes and proximal tubules in kidneys [97].

The ABC drug efflux transporter proteins P-gp, BCRP are expressed at the apical side of the enterocytes, where they are responsible for ATP-mediated extrusion of compounds back into the lumen of the intestine [100,101]. P-gp and BRCP are also expressed in canalicular membranes of hepatocytes where they function to transport compounds into the bile duct; they are also expressed in the apical membrane of the renal proximal tubules and in the endothelial cells of the blood brain barrier [80,84,85]. MRP1 is expressed on the basolateral membrane of the enterocytes and is involved in the transport of compounds into the portal vein as well as the apical membrane of the blood-brain endothelial cells [97,102,103,104]. MRP2 has been found in the apical membrane of enterocytes, hepatocytes and renal proximal tubules [102,105]. The expression of all four transporters has been described extensively for both haematological and solid tumours [100].

### 3.2. Transporter Proteins in ADME and TKI MDR in Cancer

**Figure 2:**
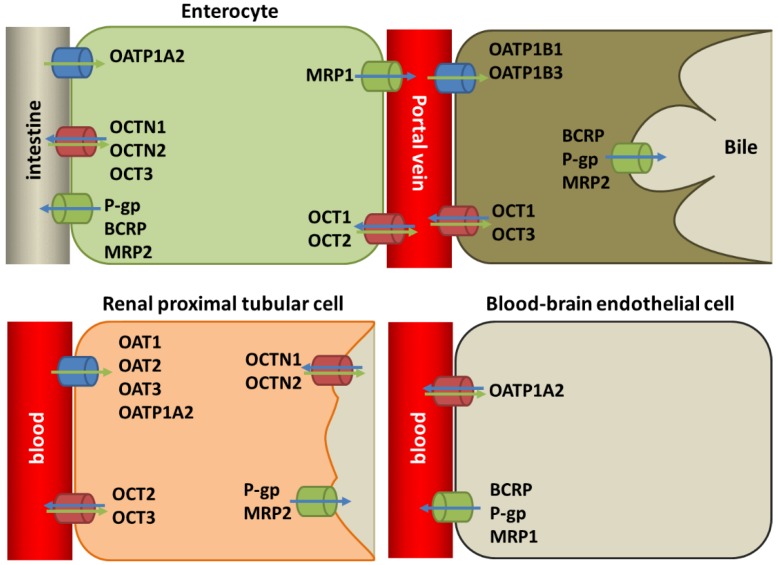
Overview of transporters and their tissue localization relevant for TKI ADME, categorized according to influx, efflux of bidirectional transporters. Arrows indicate direction of drug transport.

Together, these transporters likely play a key role in ADME of many TKIs, as they exist at multiple barriers (Figure 2). They have the potential to affect drug exposure of administered TKIs during treatment [12]. Relevant transporters are highly expressed in liver, the main organ involved in metabolism and clearance for TKIs. Interpatient variation in TKI transporters and also Cytochrome P450 expression are other known factors impacting TKI drug exposure and adverse effects [106]. This suggests that different patient subpopulations might exist that would respond differently according to their genotypes and phenotypes. It has already been suggested that monitoring drug-levels in blood is a strategy that allows for optimizing administered doses for optimal drug exposure. In addition, identification of subpopulations with a relevant phenotype could be used for personalized medicine using screening-based drug selection, allowing for a more optimal response rate in TKI-based drug treatment [11].

In contrast to the ABC family of efflux transporters, SLC22 and SLCO family members of influx transporters are less commonly implicated in multidrug resistance in cancer cells. Similarly, in stem cells, ABC drug efflux transporters rather than influx transporters constitute a phenotypic marker associated with multidrug resistance [107,108]. In addition, ABC transporters display a broad substrate affinity for various TKIs, while SLC transporters seem to have a much more specific range of drug substrates (Table 1).

### 3.3. Molecular Alterations in Transporter Proteins Affecting Drug Resistance to TKIs

#### 3.3.1. Genetic Polymorphisms

While genetic polymorphisms of TKI transporters have been reported to impact drug treatment in a variety of ways, the results are conflicting [12]. For example, in vitro data showed that the 1199G > A polymorphism in *ABCB1* (P-gp) was associated with increased efflux activity for Imatinib, Nilotinib and Dasatinib, when compared to wild type genotype [109]. However, another in vitro study reported that different P-gp haplotypes had no significant influence and that transporter expression levels dominantly influenced TKI activity [110]. The common P-gp haplotype (1236C > T-2677G > T-3435C > T) has also been reported to confer a higher efflux of imatinib compared with wild-type but the opposite was found in another study in which the TTT haplotype was associated with decreased resistance along with comparable expression levels between different *ABCB1* haplotypes [111]. Several factors indicated by Skoglund, et al. [92] may explain these apparent discrepancies, the primary one being that the analysis focused on fold change in IC50s compared to the empty vector rather than a direct comparison between wild type and polymorphic variants. The results point to differences in IC50 values between ABCB1 polymorphic variants and wild type, but expression levels appeared to outweigh the effect of polymorphisms on affinity when evaluated separately. This at least indicates that molecular alterations in P-gp can affect TKI efficacy, but data are not conclusive. Examples include the resistance against bosutinib in PgP-overexpression K562DOX cells [57], while P-gP overexpression has also been found in ceritinib-resistant cells [107].

Similar issues were noted when these *ABCB1* polymorphisms were studied in patient samples. In fact, in clinical studies, the 1236C > T-2677G > T-3435C > T is considered to be the most relevant polymorphism displaying a significant association with survival, response or toxicity for imatinib, sunitinib and sorafenib [12]. A larger meta-analysis of the clinical data is to be recommended to fully define associations with different polymorphisms in P-gp to include this determinant in personalized medicine approaches for TKI treatment. In addition, extended in vitro functional studies are warranted to pinpoint the impact of P-gp polymorphisms on efflux rates and retention of individual TKIs.

With respect to *ABCG2* (BCRP) polymorphisms, various individual SNPs were identified impacting affinities for various TKIs compared to wild type [112]. The SNPs: 34G > A, 421C > A, 623T > C, 886G > C, 1574T > G, and 1582G > A introduced a decreased ABCG2 transporter affinity for imatinib, dasatinib, nilotinib, and bosutinib, whereas 1582G > A did not significantly decrease BCRP’s affinity for nilotinib. This study also reported that a 623T > C SNP gave rise to a reduced expression of *ABCG2*, resulting in increased sensitivity to all tested drugs to a level of empty vector controls. Consistent with in vitro data, clinical studies showed that 34A > G and 421G > A SNPs were associated with reduced ABCG2 drug resistance levels [12,113]. Notably, the 34A > G SNP is common in Caucasian populations, while 421G > A is more common in Asian populations [114]. Functionally, the 421G > A SNP has been associated with reduced expression due to its location with the ATPase functional domain of BCRP, thus interfering with transporter activity [115,116]. The 34A > G SNP does not seem to affect expression levels of *BCRP* transcripts, but might be implicated in affecting alternatively spliced variants of the *ABCG2* gene, resulting in the reduced expression of ABCG2 in the liver [12,116,117].

Influx transporters of the SLC22 and SLCO family are less often implicated in multidrug resistance, but still have clinical relevance for drug resistance and ADME, when TKIs are substrates and the tissue expresses the transporters. OCT1 has been implicated in liver cancer and is often downregulated in liver cancer [68]. Several *OCT1* polymorphisms have been described that prevent proper expression, a 262T deletion and 181CG > T insert deletion. These polymorphisms result in truncated proteins unable to reach the plasma membrane [51]. *OCT1* polymorphisms have also been investigated in relation to imatinib treatment for CML [118]. Both the 1258ATG deletion and the 480G > C SNP can increase the risk of imatinib treatment failure as a result of significantly decreased imatinib uptake [11,100]. The 480G > C SNP is also associated with increased risk for treatment failure. Polymorphisms in other transporter proteins related to TKI uptake have been studied to a lesser extent [12]. An overview of the relevant polymorphisms of TKI influx and efflux transporters and their impact on TKI resistance is summarized in Table 2.

#### 3.3.2. Other Molecular Alterations Affecting Transporter Expression and Function

Beyond genetic polymorphisms, transcriptional and post-transcriptional regulation of transporter expression can also play a role in acquired drug resistance to TKIs [12].

Transcriptional regulation of transporter proteins, in particular those of the ABC family, seems to be regulated by cancer-related signaling proteins and pathways, including p53 and Ras [119]. Other regulatory pathways involved in the induction of ABC transporters include the MED1, which has been linked with drug resistant and aggressive phenotypes, and MEF-1, a regulator of P-gp expression, which has been detected in HL60 drug-resistant leukemia cell line [120,121]. It has also been shown that ABC transporter expression is induced by stress conditions, e.g., inflammation, hypoxia and also the presence of carcinogens and chemotherapeutic drugs [119].

Lastly, epigenetic factors and microRNAs are also known to play a role in the regulation of ABC transporters [119,122]. For example, the microRNAs miR-451 and miR-27a have been linked to overexpression of P-gp in multidrug resistant cell lines, conceivably by inhibition of factors that downregulate P-gp and other ABC transporter expression [123]. There are also miRNA involved in the direct downregulation of ABC transporters, suggesting that mutations of these microRNAs contribute to overexpression [122]. Finally, hypomethylation in *ABC* gene promoters and acetylation CpG island has been shown to result in upregulation of ABC transporter expression [124,125].

## 4. Lysosomal Sequestration of TKIs as Mechanism of Drug Resistance

### 4.1. Discovery of Lysosomal Sequestration of Protein Kinase Inhibitors

Recently, lysosomal sequestration has been recognized as a novel mechanism of resistance to TKIs [15]. Drug sequestration in subcellular compartments has previously been reported as a modality to confer high levels of mitoxantrone resistance in cancer cells [127]. Currently, it is being acknowledged that several anticancer drugs, including a series of TKIs, harbour biophysical properties of hydrophobic weak base molecules, which can be entrapped in the acidic milieu within lysosomes [128]. Because of the potential therapeutic implications, there is an increasing interest to understand the role of lysosomal sequestration in conferring drug resistance to individual TKIs [129]. Lysosomal sequestration of TKI was clearly linked to resistance for imatinib and sunitinib [15,130], while evidence is emerging for gefitinib and lapatinib as well [131]. Lately, several reviews have elaborated on the role of lysosomes contributing to cancer cell drug resistance [132,133,134,135].

### 4.2. Mechanisms of Drug Sequestration and the Role of Transporters

The sequestration of drugs in lysosomes induces drug resistance, as drugs are withheld to reach their cytosolic or nuclear targets [134]. For TKIs with a hydrophobic weak-base biophysical motif, entrapment within the lysosomes due to the protonation abolishes their ability to diffuse back through the lysosomal membrane into the cytosol (Figure 1). In addition, lysosomal drug sequestration induces the expression of genes involved in lysosomal biogenesis [14,136]. The master regulator in this process is Transcription Factor EB (TFEB), which promotes transcription of autophagic and lysosomal genes [137]. TFEB responds to the deprivation of growth signaling and extra-cellular nutrients, but also to stressors on the cell’s lysosomes. The activity of TFEB is mediated by mTORC1, a complex involved in energy sensing and protein synthesis regulation, which phosphorylates TFEB resulting in retention of TFEB in the cytoplasm via interaction with 14-3-3 [138]. Beyond this, accumulation of drugs in the lysosome induces membrane fluidization and permeabilization via MCOLN1 (a lysosomal membrane bound cation channel), causing the release of Ca+ and activation of TFEB translocation to the nucleus [139]. As a response, TFEB activates the CLEAR (Coordinated Lysosomal Expression and Regulation) gene network involved in lysosomal biogenesis [140]. Lastly, drug sequestration by lysosomes also triggers exocytosis via the association of lysosomes with microtubuli and fusion with the plasma membrane, effectively excreting the lysosomal content including the sequestered drugs.

While conceivably hydrophobic drugs such as TKIs can passively cross the lysosomal membrane, there is evidence that transporter proteins expressed on the lysosomal membrane have a marked enhancing effect upon lysosomal sequestration (Figure 1). In fact, transporters such as P-gp, and also ABCA3, have been linked to lysosomal-mediated multidrug resistance [130,141]. Thus, ABC transporters can contribute to TKI resistance both by extrusion of drugs over the plasma membrane and by facilitating accumulation into lysosomes/multivesicular bodies. It is possible that transporters associated with the lysosomal membrane present a more efficient mechanism of lysosomal sequestration of drugs than passive diffusion. This notion is supported by observations that intracellular ABCA3 increases the sequestration capacity of lysosomes for Imatinib in CML cells in vitro, and constitutes an indicator of poor prognosis in AML patients [130,142]. Notably, also intracellular P-gp expression in leukemia cells was more strongly correlated with their drug resistant phenotype than plasma membrane localized P-gp, thus suggesting that sequestration is potentially a more effective means of cellular drug distribution than efflux via membrane associated transporters [143].

## 5. Overcoming Transport- and Lysosome-Mediated (Multi) drug Resistance to TKIs

### 5.1. Overcoming Transport-Mediated (Multi)drug Resistance to TKIs

In a clinical setting, the main approach in dealing with treatment resistance is switching to a different drug. This is effective when resistance involves a particular molecular alteration in the drug target, as is the case with for example T790M mutation in EGFR. However, to overcome resistance due to drug influx and efflux transporters, different strategies are required. It is important to distinguish between innate expression of transporters in tissue types involved in ADME and the expression of transporters in the cancer cell, which is likely to be acquired, especially if it concerns instances of multidrug resistant phenotypes. For example, Imatinib is transported into the cell via OATP1A2 (Table 1); the expression of this influx transporter in cancer cells would in theory allow for a greater drug exposure, but this transporter is also expressed in renal tissue (Figure 2), which would facilitate the elimination of Imatinib out of blood circulation. The expression in normal cells and the tumour has to be considered when these factors are planned for the purpose of personalized medicine.

As previously stated, both molecular polymorphisms and expression are relevant factors in determining the effect upon the pharmacokinetics of certain drugs. This has not only implications for drug response but also potential toxicity, when for example clearance via liver or kidney is impaired resulting in overexposure during treatment. Transporter expression is likely to have a general predictive value when it concerns substrates. It has to be determined whether particular influx or efflux transporters are overexpressed or not in patients, either in general or in a particular type of cancer. This data may help to pre-select patients that will likely respond better to certain drugs or not, or whether higher or lower doses are required to adjust for particular ADME-related effects. Innate polymorphisms, on the other hand, are likely to have clearer rate limiting implications because they can directly alter drug affinity and transporter function. They are also a much more practical target for pre-treatment screening since such polymorphisms can easily be detected using non-invasive genetic screening of patients. There is evidence that particular genotypes present a predictive biomarker (Table 2), but there is a lack of validation of the relation between particular transporter polymorphisms and their effect upon treatment outcome for particular TKIs. Validated treatment can be designed according to the patient genotype. Alternatively drugs can be developed that are optimized for particular polymorphisms as a means to circumvent affinity of drug efflux transporters.

One interesting aspect of transporter proteins is the discovery that TKIs are also able to inhibit some transporters [48,49,50,51,144,145,146]. Many transport inhibitors have been developed since the ABC transporters are a common mechanism of resistance for many different drugs, including TKIs. These compounds have encountered problems such as severe toxicity and interactions with other drugs that severely limit their potential use in a clinical setting [147,148]. Several compounds such as Ko143 proved to be excellent tools for in vitro research, but did not proceed to the clinic. Compounds such as elacridrar, verapamil, zosuquidar and FTC moved to the clinic but were not able to reverse drug resistance, often because they did not reach effective drug levels in the patient, because of intrinsic toxicity (reviewed in [147,149]. Most TKIs, including those in clinical use for over a decade like Imatinib and Gefitinib, can specifically block ABC efflux transporter function at non-toxic concentrations. These drugs can reverse drug resistance and act as chemo sensitizers, showing increased intracellular drug accumulation when transporters are inhibited by TKIs. It is possible that homology between ATP binding sites of both the intended TK target and transporter acts as the main mechanism of action, but there is no concrete evidence that supports this. Actually a broad range of TKIs are able to block ABC transporter function in a dose-dependent manner.

While there is ample research on this potential role in vitro, there are less in vivo studies or otherwise results that describe the overall effect of using a TKI as a ABC transport blocker [150]. One of the major reasons why experiments in cell cultures cannot serve as a model is the effect of these blockers on ADME resulting in potential toxicity, since transporters are widely expressed in important tissues like the liver and the intestines (Figure 2). Several in vivo studies have recently been published. In nude mice carrying human tumour xenografts, using TKIs in combination with other anti-cancer drugs to reverse MDR no additional toxicity was observed [144,151,152,153]. This indicates that combination therapy with TKI to reverse MDR could be a viable therapeutic strategy without the toxicity of earlier efflux pump inhibitors. However, more comprehensive studies are needed. At this moment, no clinical trials are ongoing that aim at investigating this strategy [154]. There are also many novel TKIs being developed included in Table 1 that still need to be investigated as transporter substrates. In addition, it might be of interest to determine whether TKIs can be developed with the aim to target important transporters in a dose-dependent manner. This can be applied for TKIs and other drugs to reverse ABC transporter-induced drug resistance.

### 5.2. Overcoming Gut Epithelial Transport Mediated Resistance

Since TKIs are administered orally, their gut uptake is an important factor in their efficacy. Surprisingly few studies have been performed to characterize their bioavailability. From the studies using a real test for bioavailability (comparing IV administration with oral at a similar dose), it appeared that oral bioavailability is rather poor. The major problem for such studies is the poor solubility of most TKIs (Table 1), precluding IV administration. Despite the poor bioavailability, almost all TKIs are administered orally. Using a CaCo2 gut epithelial transwell system [155] we characterized the potential gut transfer for several TKI. For most TKIs, the apical (equivalent to the luminal side of the enterocyte)-basolateral (equivalent to the blood circulation) was lower than the basolateral-apical transfer, leading to an efflux ratio >1 explaining the low bioavailability. However, although bioavailability was low, it was still considered to be sufficient for most widely used TKIs to be administered orally. It was observed that the gut epithelial transfer improved with the addition of protein, which possibly plays a role in transfer. Indeed, for several TKIs, it has been observed that bioavailability improves with the addition of food [156]. A poor gut transfer seemed to be associated with a poor cellular uptake, mediated by (among others) OCTs [157]. Therefore, transport properties should be optimized before introducing a drug into the clinic, for which the CaCo-2 system can be considered as an essential screening test.

### 5.3. Overcoming Lysosome-Mediated (Multi)drug Resistance to TKIs

Since lysosomal sequestration of drugs represents a mechanism of drug resistance, it is interesting to target lysosomes or lysosomal membrane proteins to overcome such a resistance mechanism. In addition to overcoming drug resistance, targeting lysosomes has been suggested to be a therapeutic strategy on its own [132]. Lysosomes play a role in cell death mechanisms and they contain proteases and other proteins like cathepsins, which when released can induce apoptosis. Lysosomal-membrane permeabilization is required, but this involves the use of drugs to create a rupture in the lysosomal membrane. Another strategy is via the inhibition of lysosome-mediated autophagy by blocking autophagic proteins or increasing the pH of the lysosomes (in combination with another drug), eliminating their ability to sequester drugs through ion trapping [158].

Two possibilities exist: (1) prevent the accumulation of TKIs in the lysosome, or (2) disrupt the lysosomal membrane leading to an efflux of TKIs. Most strategies focus on the first option, using five major categories of agents [159] as summarized in Table 3. Chloroquine (CQ) is a drug that has been established as an anti-malarial agent, which accumulates within the lysosomes and acts as a de-acidification agent and autophagy inhibitor. Although the exact mechanism by which CQ achieves this is still unclear, it may be used to prevent other drugs from accumulating within the lysosome or to target a lysosomal protein. The analogue Hydroxychloroquine (HCQ) has been extensively tested in clinical trials and has shown some potential as an anti-autophagy agent, but these studies also showed some bottlenecks in terms of effective dosage, combined therapy, and dose-limiting toxicity. Other derivatives of CQ are being developed and investigated in order to discover analogues that have characteristics more suitable as anti-cancer agents. Drugs for other targets are still undergoing preclinical studies. These include V-ATPase inhibitors, an important enzyme that acidifies lysosomes during biogenesis, which can potentially inhibit autophagy and induces apoptosis [160]. These are often found in microorganisms, usually bacteria. Inhibitors of acid sphingomyelinase (ASM), another lysosomal membrane protein (LMP) with reduced expression, can destabilize the lysosomal membrane and cause membrane permeabilization. A recent study demonstrated the ability of such drugs to prevent lysosomal sequestration and enhance the cytotoxicity of TKI inhibitors [161]. One example of such a drug is bafilomycin, which was shown to be able prevent lysosomal sequestration but is too toxic for clinical use [15]. Another potential target is Heat shock protein 70 (HSP70), which is responsible for maintaining lysosomal membrane integrity and promotes metastasis. Its upregulation is correlated with poor prognosis. 2-Phenylethynesulfonamide (PES) or Pifitrin-μ, a small-molecule HSP70 inhibitor has shown anti-tumour activity through blockade of autophagy and induction of apoptosis through cathepsin release [162]. Inhibition of cathepsins is also suggested, but cathepsins are only oncogenic when released extracellularly, which is mediated through lysosomal exocytosis [163,164].

Up to now, no effective drug has been identified that can disrupt the lysosomal membrane after accumulation of the drug in the lysosome. An interesting approach was reported for acridones, depending upon the photodestruction of lysosomes containing accumulated Imidazoacridone [165]. The same principle was used for sunitinib [166]. This novel approach needs to be extended for other TKIs as a possibility to bypass multidrug resistance. A major hurdle for this elegant approach is the use of an activating light source in vivo.

Another approach consists of evaluation of the physico-chemical properties (see Table 1) of a TKI before introduction into the clinic, and either use these properties to optimize drug delivery or modify the drug to prevent lysosomal accumulation. For this purpose, the TKIs were classified as acid, neutral or basic, while we also analyzed the charge at several pH (Table 1; [52]). For a panel of 28 TKI, their potential accumulation in lysosomes was analyzed as well as cellular distribution using LC-MS-MS) [167,168]. There was a large variation between these TKI in the extent of lysosomal accumulation, in which a high lysosomal accumulation was found for both alkaline (sunitinib) and acid (tivantinib) TKIs. However, lysosomal accumulation seemed to be related to the theoretical pKb (high pKb, more accumulation). Using a biomimetic analysis [169] the physico-chemical properties seemed to predict accumulation in the brain (with a high accumulation for basic TKI), while the plasma to brain ratio seemed to be related to lysosomal accumulation as well. Data analyzed using an immobilized artificial membrane system (IAM) [168], showed that the IAM values might be able to predict lysosomal accumulation. 

## 6. Concluding Remarks

Recently a paper was published discussing the role of the P-gp and BCRP efflux transporters in MDR [171]. This paper suggests that while research on efflux transporters as a therapeutic was halted given the lack of positive results, renewed interest was warranted given the development of new techniques and their role in the ADME of drugs. They propose that, while blocking efflux transporters is not an effective strategy to improve therapy response, they can still serve as predictive markers for therapy response in patients that overexpress one of the ABC transporters. As drug transporters are ubiquitously expressed in epithelial and endothelial cells, blocking transporters will affect drug availability. However, as current research suggests, TKI-based blocking of efflux transporters might constitute a non-toxic strategy that could represent a more viable method. However, this requires further investigation.

Small molecule TKIs are increasingly being used in the treatment of different types of cancer but drug resistance also forms an important bottleneck in their efficacy as an anti-cancer drug. Targeting lysosomal sequestration might also constitute an alternative strategy to reverse multidrug resistance, but this strategy also suffers from toxicity issues. As new drugs targeting lysosomes are also being developed, it will be interesting to see whether targeting lysosomes can restore drug sensitivity in multidrug resistant cancer. Accumulation of drugs in lysosomes can also be considered as an opportunity, a “Trojan horse”, since the release of the drugs from the lysosomes will lead to an immediate cell death [165,166]. Although photosensitization is an elegant approach, it suffers from practicality, especially in solid tumors. Approaches with drugs that can specifically disrupt the lysosomal membrane, would be an excellent alternative.

It would also be of interest to see whether multidrug resistant cancer cells also develop resistance against these strategies. This might occur via redundant systems such as upregulation of an alternative efflux transporter or other LMPs. When these preclinical studies prove the potential of these methods to eliminate the multidrug resistance phenotype, it might be possible to improve treatment for a wide range of patients who stop responding to TKIs as well as other drugs.

Other possibilities consist of the use of other delivery systems for TKIs such as nano-particles loaded with various TKIs [172]. This will not only bypass resistance due to either limited influx or increased efflux, but will also offer the possibility to load more appropriate TKIs in one nanoparticle. This will greatly extend the possibilities of combinations, both of one TKI with another TKI, but also with a conventional drug or with an siRNA. In this approach, metabolism by Phase 1 or 2 enzymes can also be bypassed, as well as poor pharmacokinetics due to the limited solubility of the TKIs.

## Figures and Tables

**Table 1:** Overview of approved TKIs and several promising TKIs at various stages in development. Their molecular structure, physicochemical properties and molecular target, as well as their interaction with various influx and efflux transporters are shown. A (1) or (0) indicates whether or not a TKI is likely to be charged in an aqueous solution. The note (+) indicates a TKI is a substrate, (−) indicates that the TKI is confirmed not a substrate, and (*) or a blank spot indicates that there is no data available on the interaction between respective TKI and transporter.

Drugs	Molecular Structure (SMILES) ^1^	pKa/pKb (Predicted ChemAxon)	LogP (Predicted by ChemAxon)	Physiological Charge	logP	Molecular Target	Transporter Subtrate							References
							OATP1A2/1B1/1B3	OAT1/2/3	OCT1/2/3	OCTN1/2	P-gp	BCRP	MRP1/2	
Afatinib	CN(C)C\C=C\C(=O)NC1=C(O[C@H]2CCOC2)C=C2N=CN=C(NC3=CC(Cl)=C(F)C=C3)C2=C1	12.49/8.81	3.76	1	3.59	EGFR^mut^, ERBB2/4					−	+	+	[12]
Alectinib	CCC1=CC2=C(C=C1N1CCC(CC1)N1CCOCC1)C(C)(C)C1=C(C3=C(N1)C=C(C=C3)C#N)C2=O	12.18/6.97	5.59	1	4.15	ALK	*/−/−				−	−		[56]
Allitinib	C=CC(NC1=CC2=C(NC3=CC=C(OCC4=CC=CC(F)=C4)C(Cl)=C3)N=CN=C2C=C1)=O	14.45/3.99	5.77	0		EGFR1, ErbB2 (HER2)								
Anlotinib	COC1=CC2=C(OC3=CC=C4NC(C)=CC4=C3F)C=CN=C2C=C1OCC1(N)CC1	16.65/9.39	3.35	1		VEGFR1/2/3, FGFR1/2/3/4, PDGFRα/ß,cKIT								
Apatinib	CC1=C([NH2+]C2=C(C(NC3=CC=C(C4(CCCC4)C#N)C=C3)=O)C=CC=N2)C=NC=C1.CS([O-])(=O)=O	no predicted data available	1			VEGFR,ROS-1,RET					+	+		
Avitinib	CN1CCN(C2=C(F)C=C(NC3=NC(OC4=CC(NC(C=C)=O)=CC=C4)=C(C=CN5)C5=N3)C=C2)CC	12.6/7.25	3.88	0		EGFR^WT,T790M^								
Axitinib	[H]\C(=C(\[H])C1=CC=CC=N1)C1=C2C=CC(SC3=CC=CC=C3C(O)=NC)=CC2=NN1	7.73/5.14	5.01	1	4.2	VEGF, PDGF	*/+/+				+	−		[12,52]
AZD3759	COC(C(OC(N1CCN(C)C[C@H]1C)=O)=C2)=CC3=C2C(NC4=CC=CC(Cl)=C4F)=NC=N3	13.81/−7.1	4.03	0		EGFR								
BGB-283	O=C1NC2=NC=CC(OC3=CC([C@]4([H])[C@]([C@@H]4[C@@H]5NC6=CC=C(C(F)(F)F)C=C6N5)([H])O7)=C7C=C3)=C2CC1	no predicted data available		0		BRAF, EGFR								
Bosutinib	COC1=CC(NC2=C(C=NC3=CC(OCCCN4CCN(C)CC4)=C(OC)C=C23)C#N)=C(Cl)C=C1Cl	15.48/8.43	4.09	1	5.4	Bcr-Abl, Src					+			[57]
Brigatinib	COC1=CC(=CC=C1NC1=NC=C(Cl)C(NC2=CC=CC=C2P(C)(C)=O)=N1)N1CCC(CC1)N1CCN(C)CC1	12.88/8.54	3.66	1	4.6	ALK, EGFR	*/−/−	−/*/−	−/−/*		+	+		[58]
Cabozantinib	COC1=CC2=C(C=C1OC)C(OC1=CC=C(NC(=O)C3(CC3)C(=O)NC3=CC=C(F)C=C3)C=C1)=CC=N2	13.46/5.9	4.66	0	5.4	c-Met, VEGFR2, AXL, RET							/+	[59]
Canertinib	FC1=C(Cl)C=C(NC2=NC=NC3=CC(OCCCN4CCOCC4)=C(NC(=O)C=C)C=C23)C=C1	12.54/6.87	3.9	0	3.65	EGFR						+		[48]
Capmatinib (INC280)	O=C(NC)C1=CC=C(C2=NN3C(N=C2)=NC=C3CC4=CC=C5N=CC=CC5=C4)C=C1F	12.77/4.55	2.96	0		c-Met								
Ceritinib	CC(C)OC1=C(NC2=NC=C(Cl)C(N2)=NC2=CC=CC=C2S(=O)(=O)C(C)C)C=C(C)C(=C1)C1CCNCC1	11.58/10.07	5.81	1	5.03	ALK	*/−/*	*/−/*	−/*/*		+	+/−		[60]
Crizotinib	C[C@@H](OC1=CC(=CN=C1N)C1=CN(N=C1)C1CCNCC1)C1=C(Cl)C=CC(F)=C1Cl	Unknown/10.12	3.57	1	4.73	ALK, ROS1	*/+/+				+	−		[12,52]
CT-707	O=S(C1=C(NC2=C3C(NCC3)=NC(NC4=CC=C(N5CCC(N6CCN(C)CC6)CC5)C=C4OC)=N2)C=CC=C1)(NC(C)C)=O	10.24/8.53	3.59	0		ALK, FAK, PyK2								
Dacomitinib	H]\C(CN1CCCCC1)=C(\[H])C(O)=NC1=C(OC)C=C2N=CN=C(NC3=CC(Cl)=C(F)C=C3)C2=C1	−0.22/14.91	4.32	1	4.4	EGFR								
Dasatinib	CC1=NC(NC2=NC=C(S2)C(=O)NC2=C(C)C=CC=C2Cl)=CC(=N1)N1CCN(CCO)CC1	8.49/7.22	3.82	1	2.24	Bcr-Abl, Src	*/−/+		−/−/−		+	+		[12,52]
Defactinib	CNC(=O)C1=CC=C(NC2=NC=C(C(NCC3=C(N=CC=N3)N(C)S(C)(=O)=O)=N2)C(F)(F)F)C=C1	12.63/4.03	0.75	0	−0.74	FAK								
Dovitinib	CN1CCN(CC1)C1=CC=C2N=C(NC2=C1)C1=C(N)C2=C(NC1=O)C=CC=C2F	8.56/7.87	1.35	1	1.59	FGFR1/3, VEGFR, PDGFR, FLT3, c-KIT					−	−		[61]
Ensartinib	ClC1=CC=C(F)C(Cl)=C1[C@@H](C)OC2=CC(C(NC3=CC=C(C(N4C[C@@H](C)N[C@@H](C)C4)=O)C=C3)=O)=NN=C2N	13.82/8.00	3.95	0		ALK								
Erlotinib	COCCOC1=CC2=C(C=C1OCCOC)C(NC1=CC(=CC=C1)C#C)=NC=N2	16–14/4.62	3.2	0	2.39	EGFR		*/−/+	−/+/*		+	+		[12,52]
Famitinib	CCN(CC)CCN1CCC2=C(C(C)=C(N2)\C=C2/C(=O)NC3=CC=C(F)C=C23)C1=O	11.46/9.01	2.68	1		VEGFR								
Fruquintinib	CNC(=O)C1=C(C)OC2=CC(OC3=NC=NC4=CC(OC)=C(OC)C=C34)=CC=C12	14.99/2.57	2.64	0		VEGFR								
Gefitinib	COC1=C(OCCCN2CCOCC2)C=C2C(NC3=CC(Cl)=C(F)C=C3)=NC=NC2=C1	16.11/6.85	3.75	0	4.11	EGFR	*/*/+		−/−/*		+	+		[12,52]
Ibrutinib	NC1=NC=NC2=C1C(=NN2[C@@H]1CCCN(C1)C(=O)C=C)C1=CC=C(OC2=CC=CC=C2)C=C1	19.7/6.58	3.63	0	3.6	BTK								
Icotinib	C#CC1=CC=CC(NC2=NC=NC3=CC4=C(OCCOCCOCCO4)C=C23)=C1	Unknown/4.62	3.03	0		EGFR								
Imatinib	CN1CCN(CC2=CC=C(C=C2)C(=O)NC2=CC(NC3=NC=CC(=N3)C3=CN=CC=C3)=C(C)C=C2)CC1	12.45/8.27	4.38	1	2.48	Bcr-Abl	+/−/+	−/−/−	+/−/−	*/+	+	+	+/*	[12,52]
Lapatinib	CS(=O)(=O)CCNCC1=CC=C(O1)C1=CC2=C(C=C1)N=CN=C2NC1=CC(Cl)=C(OCC2=CC(F)=CC=C2)C=C1	15.99/7.2	4.64	1	5.14	HER2/neu, EGFR					+	+		[12,52]
Lenvatinib	COC1=C(C=C2C(OC3=CC(Cl)=C(NC(O)=NC4CC4)C=C3)=CC=NC2=C1)C(O)=N	3.56/6.1	2.16	1	2.8	VEGFR	*/−/−	−/*/−	−/−/*		+	+		[62]
Lorlatinib	C[C@H]1OC2=C(N)N=CC(=C2)C2=C(C#N)N(C)N=C2CN(C)C(=O)C2=C1C=C(F)C=C2	19.7/5.71	1.63	0		ROS1, ALK								
Nilotinib	CC1=CN(C=N1)C1=CC(=CC(NC(=O)C2=CC(NC3=NC=CC(=N3)C3=CN=CC=C3)=C(C)C=C2)=C1)C(F)(F)F	11.86/6.3	4.41	0	5.15	Bcr-Abl	*/+/−		−/−/−		+	+		[12,52]
Nintedanib	COC(=O)C1=CC=C2C(NC(=O)\C2=C(/NC2=CC=C(C=C2)N(C)C(=O)CN2CCN(C)CC2)C2=CC=CC=C2)=C1	unknown/15.0	2.4	1	3.3	VEGFR, FGFR, PDGFR	*/−/−		*/−/*		+	−	*/−	[63]
Osimertinib	COC1=C(NC2=NC=CC(=N2)C2=CN(C)C3=C2C=CC=C3)C=C(NC(=O)C=C)C(=C1)N(C)CCN(C)C	13.64/8.87	4.49	1	3.7	EGFR^T790M^	*/−/−				+	+		[64]
Pazopanib	CN(C1=CC2=NN(C)C(C)=C2C=C1)C1=CC=NC(NC2=CC=C(C)C(=C2)S(N)(=O)=O)=N1	10.41/5.07	3.55	0	1.98	VEGFR, FGFR, PDGFR, Kit, Itk, Lck, c-Fms	*/+/+				+			[12,52]
Ponatinib	CN1CCN(CC2=CC=C(NC(=O)C3=CC(C#CC4=CN=C5C=CC=NN45)=C(C)C=C3)C=C2C(F)(F)F)CC1	11.36/8.03	4.97	1	3.1	Bcr-Abl	*/−/−		−/*/*		+	+		[12,52]
Poziotinib	COC1=C(OC2CCN(CC2)C(=O)C=C)C=C2C(NC3=CC=C(Cl)C(Cl)=C3F)=NC=NC2=C1	13.99/4.49	4.5	0		EGFR, HER2/neu, Her 4								
Pyrotinib	ClC1=CC(NC2=C(C#N)C=NC3=CC(OCC)=C(NC(/C=C/[C@@H]4N(C)CCC4)=O)C=C23)=CC=C1OCC5=CC=CC=N5	12.55/8.71	4.93	0		HER2								
Quizartinib	CC(C)(C)C1=CC(NC(=O)NC2=CC=C(C=C2)C2=CN3C(SC4=C3C=CC(OCCN3CCOCC3)=C4)=N2)=NO1	10.43/6.62	5.16	0		FLT3								
Regorafenib	CNC(=O)C1=CC(OC2=CC(F)=C(NC(=O)NC3=CC=C(Cl)C(=C3)C(F)(F)F)C=C2)=CC=N1	10.52/2.02	4.49	0	5.26	VEGFR, TIE2, PDGFR-β, FGFR1, KIT, RET, c-RAF/RAF-1, BRAF^V600E^	*/−/−				−	+		[12,52]
Rociletinib	COC1=CC(=CC=C1NC1=NC=C(C(NC2=CC=CC(NC(=O)C=C)=C2)=N1)C(F)(F)F)N1CCN(CC1)C(C)=O	13.63/3.62	4.41	0		EGFR^WT,T790M,L858R^								
Ruxolitinib	N#CC[C@H](C1CCCC1)N1C=C(C=N1)C1=C2C=CNC2=NC=N1	13.89/5.51	5.51	0	2.1	JAK1/2					−			[65]
saracatinib	CN1CCN(CCOC2=CC3=C(C(NC4=C(Cl)C=CC5=C4OCO5)=NC=N3)C(OC3CCOCC3)=C2)CC1	11.81/8.19	3.53	1	2.74	Bcr-Abl, Src			+/+/+	+/−				[66]
Savolitinib	C[C@H](N1N=NC2=NC=C(N=C12)C1=CN(C)N=C1)C1=CN2C=CN=C2C=C1	Unknown/6.67	1.07	0		c-Met								
Selumetinib	CN1C=NC2=C1C=C(C(O)=NOCCO)C(NC1=C(Cl)C=C(Br)C=C1)=C2F	6.49/5.43	3.41	0	5.55	MEK1/2					+	+		[67]
Semaxanib	CC1=CC(C)=C(N1)\C=C1/C(=O)NC2=CC=CC=C12	11.29/−2.1	2.98	0	2.87	VEGFR2								
Sorafenib	CNC(=O)C1=NC=CC(OC2=CC=C(NC(=O)NC3=CC(=C(Cl)C=C3)C(F)(F)F)C=C2)=C1	11.55/2.03	4.34	0	5.16	VEGFR, PDGFR, Raf-1, C-Raf	−/+/−	*/−/*	+/*/*	−/−	+	+	*/+	[12,48,52,68]
Sunitinib	CCN(CC)CCNC(=O)C1=C(C)NC(\C=C2/C(=O)NC3=C2C=C(F)C=C3)=C1C	11.46/9.04	2.93	1	3.15	VEGF, PDGF, c-KIT	−/+/−				+	+		[12,52]
Tamatinib	COC1=CC(NC2=NC=C(F)C(NC3=NC4=C(OC(C)(C)C(=O)N4COP(O)(O)=O)C=C3)=N2)=CC(OC)=C1OC	1.46/2.71	2.78	1		SYK								
Tandutinib	COC1=C(OCCCN2CCCCC2)C=C2N=CN=C(N3CCN(CC3)C(=O)NC3=CC=C(OC(C)C)C=C3)C2=C1	14.01/9.03	4.34	1	4.38	FLT3					+	+		[69]
Tivantinib	O=C1NC(=O)[C@H]([C@@H]1C1=CNC2=CC=CC=C12)C1=CN2CCCC3=C2C1=CC=C3	9.72/−8.6	3.27	0	3.26	c-MET								
Trametinib	CN1C(=O)C(C)=C2N(C(=O)N(C3CC3)C(=O)C2=C1NC1=CC=C(I)C=C1F)C1=CC(NC(C)=O)=CC=C1	12.6/−3.7	3.18	0	2.68	MEK	−/−		−/*/*		+	−	*/−	[70]
Tofacitinib	[H][C@@]1(C)CCN(C[C@]1([H])N(C)C1=NC=NC2=C1C=CN2)C(=O)CC#N	8.46/7.13	1.24	1	1.5	JAK			*/−/*					[71]
Vandetanib	COC1=C(OCC2CCN(C)CC2)C=C2N=CN=C(NC3=C(F)C=C(Br)C=C3)C2=C1	13.8/9.13	4.54	1	5.51	VEGFR, EGFR, RET	+				−			[12,52]
Vatalanib	ClC1=CC=C(NC2=NN=C(CC3=CC=NC=C3)C3=CC=CC=C23)C=C1	15.17/4.95	4.95	0	3.8	VEGFR								
Vemurafenib	CCCS(=O)(=O)NC1=C(F)C(C(=O)C2=CNC3=NC=C(C=C23)C2=CC=C(Cl)C=C2)=C(F)C=C1	7.17/3.2	4.62	0	4.26	BRAF^V600E^	+							[12,52]

^1^ SMILES (Simplified Molecular Input Line Entry System) are a digital notation used to represent a chemical structure in such a fashioned that it can be interpreted by computer algorithms. Statistical comparisons can then be made comparing similarities between individual structures.

**Table 2:** Overview of genetic polymorphisms relevant for TKIs.

Transporters	Genetic Polymorphism	Effects on TKIs	Reference
ABCB1 (P-gp)	1199G > A	Increased efflux activity for:	[109]
		Imatinib	
		Nilotinib	
		Dasatinib	
	1236C > T-2677G > T-3435C > T (often inherited together in linkage disequilibrium)	TTT: Increased sensitivity to	[111]
		Imatinib and decreased efflux (compared to CGC wild type)	[12]
		TTT: Increased resistance to sunitinib	
		CGT: Increased progression free survival	
		CGT: Increased toxicity for sorafenib	
ABCG2 (BCRP)	34G > A	G > A: decreased affinity and increased drug efficacy for imatinib, dasatinib, nilotinib, and bosutinib	[112]
		GG: Decreased overall survival in NSCLC patients receiving EGFR-inhibitors (gefitinib, erlotinib and icotinib) compared to other genotypes	[113]
		G > A: Increased major molecular response in imatinib	[12]
	421C > A	Reduced affinity and increased drug efficacy for imatinib, dasatinib, nilotinib, and bosutinib	[12,112]
	623T > C	Loss of expression, increased drug efficacy for imatinib, dasatinib, nilotinib, and bosutinib	[112]
	886G > C	Reduced affinity and increased drug efficacy for imatinib, dasatinib, nilotinib, and bosutinib	[112]
	1574T > G	Reduced affinity and increased drug efficacy for imatinib, dasatinib, nilotinib, and bosutinib	[112]
	1582G > A	increased drug efficacy for imatinib, dasatinib, and bosutinib	[112]
SLC22A1 (OCT1)	262Tdel	Truncated protein: reduced sensitivity for sorafenib	[68]
	181CGdelTins	Truncated protein: reduced sensitivity for sorafenib	[68]
	1258ATGdel	Increased probability of treatment failure for imatinib	[12,118]
	480G > C	Loss of sensitivity or treatment failure for imatinib	[12,126]
SLC22A4 (OCTN1)	1507C > T	TT genotype: Reduced major molecular response in imatinib	[12]
SLCO1B3 (OATP1B3)	334T > G	TT genotype: Reduced complete molecular response in imatinib	[12]

**Table 3:** Overview of candidate anti-lysosomal agents.

Drug	Class	Tested in	References
Bafilomycin A1	V-ATPase inhibitor	In vitro	[159]
Hydroxychloroquine/chloroquin	De-acidifier	clinical trials	[159]
Lys5	De-acidifier	In vivo	[159]
Manzamine A	V-ATPase inhibitor	In vitro	[160,170]
Pifitrin-μ	HSP70 inhibitor	In vivo	[162]
Light sensitive drug	Photosensitizer	In vitro	[166]
